# DHCR24 inhibitor SH42 increases desmosterol without preventing atherosclerosis development in mice

**DOI:** 10.1016/j.isci.2024.109830

**Published:** 2024-04-26

**Authors:** Xiaoke Ge, Bram Slütter, Joost M. Lambooij, Enchen Zhou, Zhixiong Ying, Ceren Agirman, Marieke Heijink, Antoine Rimbert, Bruno Guigas, Johan Kuiper, Christoph Müller, Franz Bracher, Martin Giera, Sander Kooijman, Patrick C.N. Rensen, Yanan Wang, Milena Schönke

**Affiliations:** 1 Department of Medicine, Div. of Endocrinology, and Einthoven Laboratory for Experimental Vascular Medicine, Leiden University Medical Center, Leiden 2333 ZA, the Netherlands; 2 Div. of BioTherapeutics, Leiden Academic Center for Drug Research, Leiden University, Leiden 2333 AL, the Netherlands; 3 Department of Parasitology, Leiden University Medical Center, Leiden 2333 ZA, the Netherlands; 4 Department of Cell and Chemical Biology, Leiden University Medical Center, Leiden 2333 ZA, the Netherlands; 5 The Center for Proteomics and Metabolomics, Leiden University Medical Center, Leiden 2333 ZA, the Netherlands; 6 Nantes Université, CNRS, INSERM, l’institut du thorax, F-44000 Nantes, France; 7 Department of Pharmacy, Center for Drug Research, Ludwig Maximilians Universität München, 80539 Munich, Germany; 8 Med-X institute, Center for Immunological and Metabolic Diseases, and Department of Endocrinology, First Affiliated Hospital of Xi’an Jiaotong University, Xi’an Jiaotong University, Xi’an 710061, China

**Keywords:** Cardiovascular medicine, Cellular physiology, Molecular genetics;

## Abstract

The liver X receptor (LXR) is considered a therapeutic target for atherosclerosis treatment, but synthetic LXR agonists generally also cause hepatic steatosis and hypertriglyceridemia. Desmosterol, a final intermediate in cholesterol biosynthesis, has been identified as a selective LXR ligand that suppresses inflammation without inducing lipogenesis. Δ24-Dehydrocholesterol reductase (DHCR24) converts desmosterol into cholesterol, and we previously showed that the DHCR24 inhibitor SH42 increases desmosterol to activate LXR and attenuate experimental peritonitis and metabolic dysfunction-associated steatotic liver disease. Here, we aimed to evaluate the effect of SH42 on atherosclerosis development in APOE∗3-Leiden.CETP mice and low-density lipoproteins (LDL) receptor knockout mice, models for lipid- and inflammation-driven atherosclerosis, respectively. In both models, SH42 increased desmosterol without affecting plasma lipids. While reducing liver lipids in APOE∗3-Leiden.CETP mice, and regulating populations of circulating monocytes in LDL receptor knockout mice, SH42 did not attenuate atherosclerosis in either model.

## Highlights

• DHCR24 inhibitor SH42 increases desmosterol in mice without causing hyperlipidemia
• SH42 treatment decreases hepatic lipids in hyperlipidemic APOE∗3-Leiden.CETP mice
• SH42 treatment lowers the abundance of blood monocytes in LDLr-KO mice
• SH42 treatment does not affect atherosclerosis development in mice

## Graphical abstract

**Figure:**
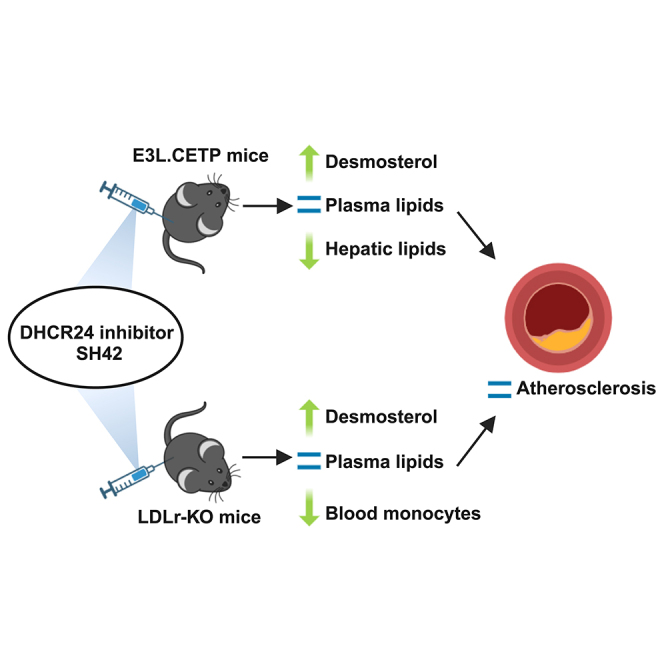

## Introduction

Cardiovascular diseases (CVDs) are the leading cause of mortality worldwide, responsible for an estimated 32% of all global deaths.^1^ The main underlying cause of CVDs is atherosclerosis, which is characterized by the buildup of lipids and immune cells inside the walls of arteries. Hypercholesterolemia drives the accumulation of low-density lipoproteins (LDL) in artery walls. LDL is taken up by monocyte-derived macrophages that turn into foam cells, resulting in fatty streaks in the intima of vessels. Foam cell generation induces the production and secretion of pro-inflammatory cytokines that further stimulate monocyte recruitment and transdifferentiation of smooth muscle cells into macrophage-like cells, supporting a chronic inflammatory response and eventually acceleration of atherosclerotic lesion development.^2^

Numerous studies have demonstrated that activating liver X receptors (LXR), crucial regulators of lipid metabolism and immune responses, protects against atherosclerosis development in animals.^3^^,^^4^^,^^5^ LXR activation in macrophages promotes reverse cholesterol transport, i.e., increases the efflux of cholesterol from macrophages toward the liver, through upregulation of the expression of macrophage genes involved in cholesterol efflux including ATP-binding cassette transporter A1 (ABCA1) and ATP-binding cassette transporter G1 (ABCG1). Accordingly, foam cell formation is reduced and inflammatory responses are suppressed.^4^^,^^6^ However, LXR activation by synthetic LXR agonists such as T0901317 and GW3965 also stimulates lipogenesis in hepatocytes via the activation of sterol regulatory element-binding transcription proteins (SREBP), which causes hepatic steatosis and hypertriglyceridemia.^7^^,^^8^^,^^9^^,^^10^^,^^11^ Accordingly, clinical trials using synthetic LXR agonists showed dyslipidemic side effects,^12^ based on which clinical programs with LXR agonists were discontinued.

Desmosterol, the last intermediate in the Bloch pathway of *de novo* cholesterol synthesis, is involved in selective reprogramming of lipid metabolism and suppression of inflammation by activating LXR target genes and inhibiting SREBP activity by binding to SREBP cleavage-activating protein in macrophages.^13^ On the contrary, desmosterol does not seem to activate LXR in hepatocytes.^14^ This implies that increasing desmosterol concentrations may be a potential safe strategy in the treatment of diseases driven by hypercholesterolemia and inflammation. We have previously generated SH42 as inhibitor of Δ24-dehydrocholesterol reductase (DHCR24), the enzyme catalyzing conversion of desmosterol into cholesterol,^15^ and showed that SH42 treatment increases desmosterol levels to enhance inflammation resolution in a peritonitis mouse model.^16^ Recently, we also demonstrated that SH42 effectively inhibits liver inflammation by reducing Kupffer cell activation and monocyte infiltration, hence preventing diet-induced hepatic inflammation and steatosis.^17^ Importantly, LXR activation through desmosterol accumulation did not result in hypertriglyceridemia in our studies. The present study aimed to investigate whether SH42 could also effectively reduce atherosclerosis development. To this end, we used APOE∗3-Leiden.CETP (E3L.CETP) mice, a well-established human-like model for lipid-driven atherosclerosis, and LDL receptor knockout (LDLr-KO) mice, a model for inflammation-driven atherosclerosis.^18^

## Results

### SH42 increases desmosterol levels in the liver and plasma without affecting plasma lipid levels in APOE∗3-Leiden.CETP mice

**Figure 1:**
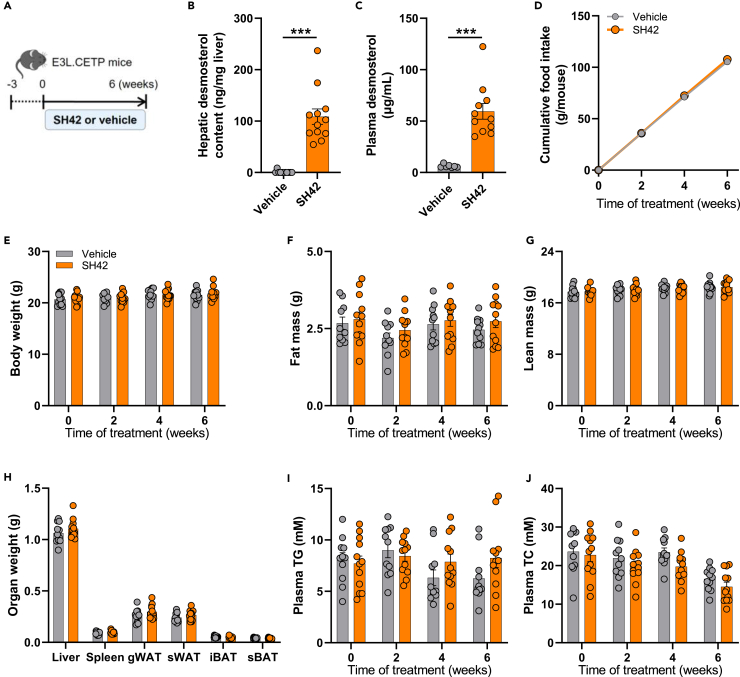
DHCR24 inhibitor SH42 increases desmosterol levels in the liver and plasma without affecting plasma lipid levels in APOE∗3-Leiden.CETP mice with 6 weeks of treatment APOE∗3-Leiden.CETP (E3L.CETP) mice were fed a Western-type diet containing 16% fat and 0.15% cholesterol and received intraperitoneal injections with either SH42 (0.5 mg/mouse) or vehicle 3 times per week (A). After 6 weeks of treatment, livers (B) and plasma (C) were collected to measure desmosterol levels. Cumulative food intake (D), body weight (E), fat mass (F) and lean mass (G) were determined every 2 weeks. After 6 weeks of treatment, the weight of various organs (H) was determined. Fasting plasma triglycerides (TG) (I) and total cholesterol (TC) (J) were measured every 2 weeks gWAT, gonadal white adipose tissue; iBAT, interscapular brown adipose tissue; sBAT, subscapular brown adipose tissue; sWAT, subcutaneous white adipose tissue. Data are shown as mean ± SEM. B, C, and E–J: n = 7–12 mice per group; D: *n* = 4 cages per group. B and C: data were analyzed by two-tailed Mann Whitney U test; H: data were analyzed by unpaired two-tailed Student’s t test; D–G, I and J: data were analyzed by two-way repeated-measures ANOVA and Bonferroni post hoc analysis. ∗∗∗*p* < 0.001.

The E3L.CETP mouse is a well-established model of human-like lipoprotein metabolism in which females develop more pronounced hypercholesterolemia and atherosclerosis than males on a Western-type diet,^19^^,^^20^ so we used female E3L.CETP mice in this study. We first evaluated the efficacy of the DHCR24 inhibitor SH42 to increase the levels of desmosterol in these mice fed a Western-type diet after 6 weeks of treatment (Figure 1A). As expected, SH42 remarkably elevated the desmosterol levels in the liver (137-fold; Figure 1B) and plasma (9.7-fold; Figure 1C). Throughout the study, SH42 treatment did not affect food intake (Figure 1D), body weight (Figure 1E), body fat mass (Figure 1F) and lean mass (Figure 1G), or the weight of metabolic organs including adipose tissue depots collected at the end of the study (Figure 1H). In line with the notion that desmosterol does not activate SREBP,^14^ treatment with SH42 did not affect plasma triglycerides (TG; Figure 1I) or total cholesterol (TC; Figure 1J). Meanwhile, a decrease in plasma TC (Figure 1J) was observed after 6 weeks compared to the baseline, regardless of treatment. This may be the outcome of self-regulation as the mice progressively get used to the cholesterol-rich diet and we have observed a similar fluctuation of plasma TC levels in previous studies with E3L.CETP mice fed a Western-type diet.^21^^,^^22^^,^^23^

### SH42 reduces hepatic lipid content in APOE∗3-Leiden.CETP mice

**Figure 2:**
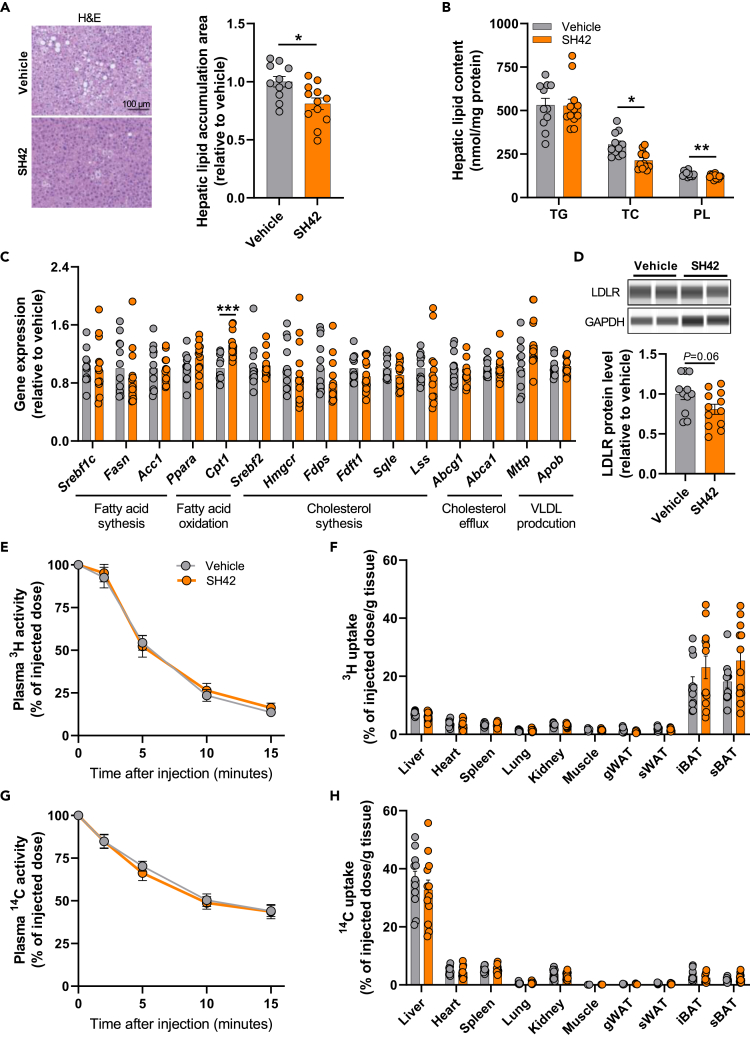
DHCR24 inhibitor SH42 reduces hepatic lipid content without affecting the plasma decay and organ uptake of triglyceride-rich lipoprotein-like particles in APOE∗3-Leiden.CETP mice After 6 weeks of treatment, the hepatic area of lipid accumulation area (A) was quantified following hematoxylin-eosin (H&E) staining and hepatic lipid content (B) was measured. The relative mRNA expression levels of genes involved in lipid metabolism were determined in the liver (C). Hepatic low-density lipoprotein receptor (LDLR) protein abundance was measured (D). Mice were injected with triglyceride-rich lipoprotein (TRL)-like particles, double-labeled with glycerol tri[^3^H]oleate ([^3^H]TO) and [^14^C]cholesteryl oleate, and the activity of ^3^H and ^14^C in plasma (E and G) and various tissues (F and H) was assessed. TC, total cholesterol; TG, triglycerides; PL, phospholipids; *Abca1*, ATP binding cassette subfamily A member 1; *Abcg1*, ATP binding cassette subfamily G member 1; *Acc1*, acetyl coenzyme A carboxylase 1; *Apob*, apolipoprotein B; *Cpt1*, carnitine palmitoyl transferase 1; *Fasn*, fatty acid synthase; *Fdft1*, farnesyl-diphosphate farnesyltransferase 1; *Fdps*, farnesyl diphosphate synthetase; *Hmgcr*, 3-hydroxy-3-methylglutaryl coenzyme A; *Lss*, lanosterol synthase; *Mttp*, microsomal triglyceride transfer protein; *Ppara*, peroxisome proliferator-activated receptor alpha; *Sqle*, squalene epoxidase; *Srebf1c*, sterol regulatory element-binding factor 1c; *Srebf2*, sterol regulatory element-binding factor 2; gWAT, gonadal white adipose tissue; iBAT, interscapular brown adipose tissue; sBAT, subscapular brown adipose tissue; sWAT, subcutaneous white adipose tissue. Data are shown as mean ± SEM. *n* = 10–12 mice per group. A–D, F, and H: data were analyzed by unpaired two-tailed Student’s t test; E and G: data were analyzed by two-way repeated-measures ANOVA. ∗*p* < 0.05, ∗∗*p* < 0.01, ∗∗∗*p* < 0.001.

Since SH42 protects against the development of metabolic disease-associated steatotic liver diseases on a high-fat high-cholesterol diet,^17^ we evaluated whether SH42 also reduces lipid accumulation in the livers of Western-type diet-fed E3L.CETP mice. While SH42 treatment did not affect liver weight (Figure 1H), histological analysis showed reduced lipid accumulation (−19%; Figure 2A). Specifically, SH42 reduced hepatic levels of TC (−30%) and phospholipids (PL; −9%) (Figure 2B). To investigate the underlying causes, we quantified the expression of hepatic genes involved in lipid metabolism. SH42 increased the gene expression of carnitine palmitoyl transferase 1 (*Cpt1*; Figure 2C) which is involved in fatty acid oxidation, while not altering markers of fatty acid synthesis, cholesterol synthesis, cholesterol efflux or very low-density lipoprotein production. Meanwhile, SH42 tended to decrease the protein abundance of hepatic LDLR (−19%; *p* = 0.06; Figure 2D), which may suggest that SH42 reduces hepatic TG-rich lipoprotein remnant uptake. To test this, we intravenously injected mice with TG-rich lipoprotein-like particles labeled with glycerol tri[^3^H]oleate ([^3^H]TO) and [^14^C]cholesteryl oleate ([^14^C]CO). However, SH42 did not affect the plasma clearance of [^3^H]TO (Figure 2E) or [^14^C]CO (Figure 2G), or the uptake of radiolabels by the various organs including the liver (Figures 2F and 2H).

### SH42 does not affect the population of circulating monocytes or atherosclerosis development in APOE∗3-Leiden.CETP mice

**Figure 3:**
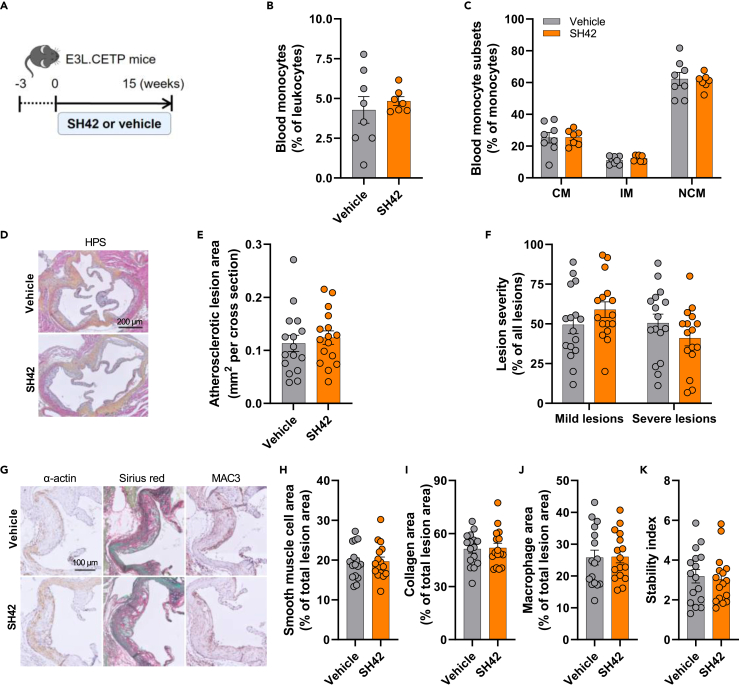
DHCR24 inhibitor SH42 does not affect the population of circulating monocytes or atherosclerosis development in APOE∗3-Leiden.CETP mice with 15 weeks of treatment APOE∗3-Leiden.CETP (E3L.CETP) mice were fed a Western-type diet containing 16% fat and 0.15% cholesterol and received intraperitoneal injections with either SH42 (0.5 mg/mouse) or vehicle 3 times per week (A). After 15 weeks of treatment, blood monocytes (B) and their subsets (C) were quantified. Hearts were collected, the aortic root valve areas were stained with hematoxylin-phloxine-saffron (HPS), and representative pictures are shown (D). The atherosclerotic lesion area (E) was determined and lesions were categorized according to lesion severity (F). The smooth muscle cell (H), collagen (I) and macrophage (J) areas of the lesions were determined by staining with an anti-α-actin antibody, Sirius Red and anti-MAC3 antibody, respectively. Representative pictures are shown (G). The lesion stability index (smooth muscle cell area and collagen area/macrophage area of the lesions) was calculated (K). CM, classical monocyte; IM, intermediate monocyte; NCM, nonclassical monocyte. Data are shown as mean ± SEM. B and C: n = 7–8 mice per group; E, F, and H–K: *n* = 16 mice per group. Data were analyzed by unpaired two-tailed Student’s t test.

To investigate whether SH42 reduces atherosclerosis development, E3L.CETP mice were treated with SH42 or vehicle for 15 weeks (Figure 3A). Throughout the study, SH42 treatment did not affect food intake (Figure S1A), body weight (Figure S1B), body fat mass (Figure S1C) and lean mass (Figure S1D), or plasma TG (Figure S1F) and TC (Figure S1G), and only slightly increased the weight of subscapular brown adipose tissue (sBAT; Figure S1E). As the macrophages in atherosclerosis lesions are primarily derived from circulating monocytes infiltrating the plaque,^24^ we evaluated the effects of SH42 on circulating monocytes. However, the relative abundance of circulating monocytes (Figure 3B) and monocyte subsets (Figure 3C) were not affected by SH42 treatment. Next, the aortic root of the heart was stained with hematoxylin-phloxine-saffron (HPS) to assess the size of the atherosclerotic lesions (Figure 3D). We did not observe obvious effects of SH42 treatment data on atherosclerotic lesion area (Figure 3E) or severity (Figure 3F). Immunohistochemical staining of specific markers expressed in the atherosclerotic plaque revealed that lesion composition was also not affected with respect to the areas of smooth muscle cells, collagen, and macrophages (Figures 3G–3J), and no difference in the lesion stability index was observed (Figure 3K). These data demonstrate that SH42 does not affect atherosclerosis development in E3L.CETP mice.

### SH42 increases desmosterol levels in the liver and plasma without affecting plasma lipid levels in LDL receptor knockout mice

**Figure 4:**
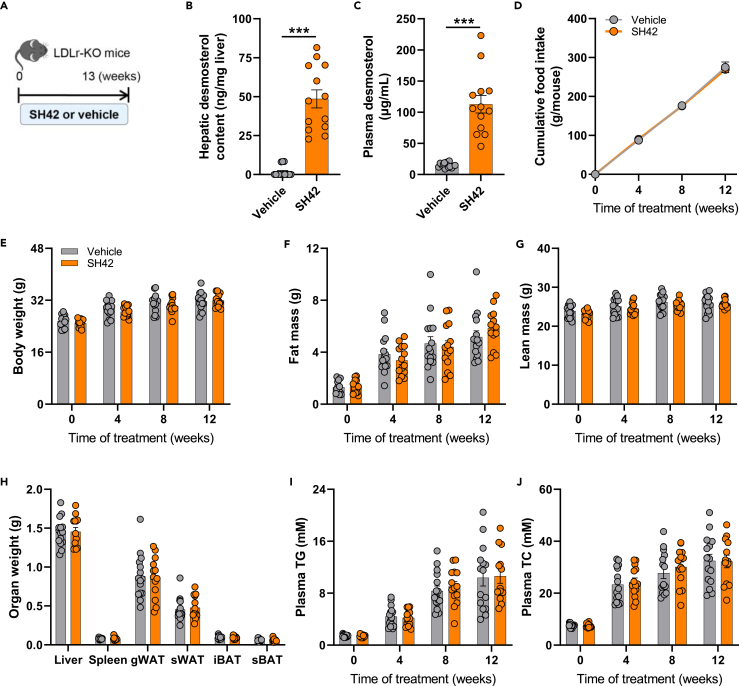
DHCR24 inhibitor SH42 increases desmosterol levels in the liver and plasma without affecting plasma lipid levels in LDL receptor knockout mice with 13 weeks of treatment LDL receptor knockout (LDLr-KO) mice were fed a Western-type diet containing 16% fat and 0.25% cholesterol and received intraperitoneal injections with either SH42 (0.5 mg/mouse) or vehicle 3 times per week (A). After 13 weeks of treatment, mice were killed, and livers (B) and plasma (C) were collected to measure desmosterol levels. Cumulative food intake (D), body weight (E), fat mass (F) and lean mass (G) were determined every 4 weeks. After 13 weeks of treatment, the weight of various organs (H) was determined. Fasting plasma triglycerides (TG) (I) and total cholesterol (TC) (J) were measured every 4 weeks gWAT, gonadal white adipose tissue; iBAT, interscapular brown adipose tissue; sBAT, subscapular brown adipose tissue; sWAT, subcutaneous white adipose tissue. Data are shown as mean ± SEM. B, C, and E–J: *n* = 13–15 mice per group; D: n = 6–7 cages per group. B and C: data were analyzed by two-tailed Mann Whitney U test; H: data were analyzed by unpaired two-tailed Student’s t test; D–G, I, and J: data were analyzed by two-way repeated-measures ANOVA. ∗∗∗*p* < 0.001.

Since atherosclerosis development in E3L.CETP mice fed a Western-type diet is mainly lipid-driven, we next assessed the effects of SH42 treatment on atherosclerosis development in LDLr-KO mice, a typical model for inflammation-driven atherosclerosis studies.^18^ Given that males develop more inflamed plaques compared to females on an atherogenic diet,^25^ male LDLr-KO mice were used in this study, and fed a Western-type diet and treated with SH42 or vehicle for 13 weeks (Figure 4A). Also in LDLr-KO mice, SH42 treatment induced a robust increase in desmosterol levels in the liver (29-fold; Figure 4B) and plasma (7.4-fold; Figure 4C) without affecting food intake (Figure 4D), body weight (Figure 4E), body fat mass (Figure 4F) and lean mass (Figure 4G), or the weight of several organs (Figure 4H). Throughout the treatment period, SH42 did not affect the plasma levels of TG (Figure 4I) or TC (Figure 4J).

### SH42 decreases circulating non-classical monocytes without affecting atherosclerosis development in LDL receptor knockout mice

**Figure 5:**
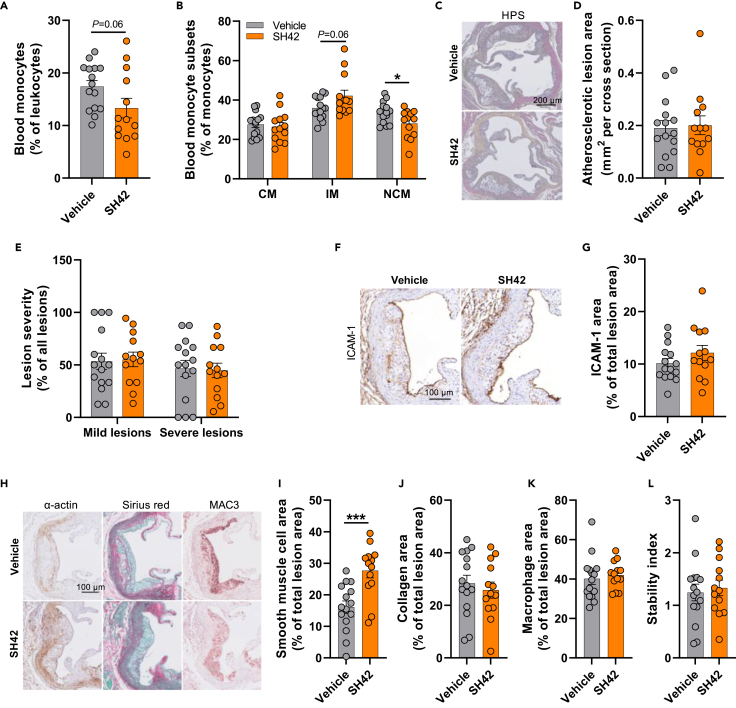
DHCR24 inhibitor SH42 decreases circulating non-classical monocytes without affecting atherosclerosis development in LDL receptor knockout mice After 13 weeks of treatment, mice were killed and the relative number of blood monocytes (A) and their subsets (B) were quantified. Hearts were collected, the aortic root valve areas were stained with hematoxylin-phloxine-saffron (HPS), and representative pictures are shown (C). The atherosclerotic lesion area (D) was determined and lesions were categorized according to lesion severity (E). Intercellular adhesion molecule 1 (ICAM-1) area (G) in lesions was stained using an anti-ICAM-1 antibody. Representative pictures are shown (F). The smooth muscle cell (I), collagen (J) and macrophage (K) areas of the lesions were determined by staining aortic root lesions with an anti-α-actin antibody, Sirius Red and anti-MAC3 antibody, respectively. Representative pictures are shown (H). The stability index (smooth muscle cell area and collagen area/macrophage area of the lesions) was calculated (L). CM, classical monocyte; IM, intermediate monocyte; NCM, nonclassical monocyte. Data are shown as mean ± SEM. *n* = 13–15 mice per group. Data were analyzed by unpaired two-tailed Student’s t test. ∗*p* < 0.05, ∗∗∗*p* < 0.001.

In LDLr-KO mice, SH42 tended to decrease the relative abundance of total circulating monocytes (−24%; *p* = 0.06; Figure 5A) and intermediate monocytes (IM, −17%; *p* = 0.06) and significantly decreased the proportion of non-classical monocytes (NCM; −17%) (Figure 5B). Nonetheless, SH42 treatment did not affect the atherosclerotic lesion area (Figures 5C and 5D) or lesion severity (Figure 5E). The area of ICAM-1 (Figure 5F and 5G) was not altered by SH42 treatment. Immunohistochemical stainings of specific markers expressed in the atherosclerotic plaque were performed to reveal the lesion composition (Figure 5H). Interestingly, SH42 treatment did increase the relative smooth muscle cell area (+72%; Figure 5I) in the atherosclerotic lesions but as the lesion collagen area (Figure 5J) and macrophage area (Figure 5K) were not affected, the lesion stability index (Figure 5L) was unchanged by SH42 treatment. These data suggest that even though SH42 did regulate the population of circulating monocytes in LDLr-KO mice, it did not attenuate atherosclerosis development.

### Genetic association of *DHCR24* variants with coronary artery disease in humans

Since DHCR24 inhibition by SH42 did not affect atherosclerosis in both mouse models, we next evaluated whether common genetic variants within the *DHCR24* locus would associate with coronary artery disease (CAD) in humans using the largest dataset published to date.^26^ When querying the entire *DHCR24* locus (chr1.p32.3, 500 kb up and downstream of *DHCR24*), we could observe variants very strongly associated with CAD (Figure S2, upper panel). However, the *DHCR24* locus encompasses the proprotein convertase subtilisin/kexin type 9 (*PCSK9*) gene (∼150 kb downstream of *DHCR24*), one of the most closely related loci with LDL cholesterol levels and CAD in humans (Figure S2, upper panel). Although functional evidence does not support the association of *Dhcr24* with atherosclerosis in mice and that no variant reaches significance (*p* = 5.0E-8) in the genomic region of *DHCR24 per se* in humans (Figure S2, lower panel), we cannot state with certainty that there is no genetic association between the *DHCR24* locus and CAD in humans.

## Discussion

LXR activation in macrophages is a potential strategy to attenuate atherosclerosis development, but currently available synthetic LXR agonists also stimulate lipogenesis in hepatocytes via the activation of SREBP,^7^^,^^8^^,^^9^^,^^10^^,^^11^ based on which their clinical development has failed. Interestingly, desmosterol has been reported to selectively activate LXR in macrophages without inducing lipogenesis.^13^^,^^14^ In the present study, we investigated the effects of desmosterol accumulation induced by SH42, a specific DHCR24 inhibitor, on lipid metabolism and atherosclerosis development in both E3L.CETP mice and LDLr-KO mice. We demonstrated that SH42 robustly increases the desmosterol levels in the liver and plasma in both mouse models, accompanied by a reduction in hepatic lipids as shown in E3L.CETP mice, and an improved regulation of circulating monocyte populations as shown in LDLr-KO mice, albeit without affecting atherosclerosis development in either model.

First, we observed that SH42 largely increases desmosterol levels in the liver and plasma. Other DHCR24 inhibitors, in addition to inducing accumulation of desmosterol, like DMHCA and triparanol, also induce accumulation of other intermediates of cholesterol biosynthesis, including zymosterol and lathosterol,^15^^,^^27^ and at least the latter has been reported to negatively correlate with the risk of CVDs.^28^ SH42 has been shown to primarily induce a robust accumulation of desmosterol.^15^ Therefore, it is convincing that the effects of SH42 in this study were from the accumulated desmosterol, instead of other sterols. With the elevated desmosterol levels, we observed a reduction in hepatic lipids as shown in E3L.CETP mice and unaltered plasma lipids in both mouse models. The fact that the increase in the endogenous LXR agonist desmosterol does not induce hepatosteatosis or dyslipidemia is in favorable contrast to the effects of synthetic LXR agonists that stimulate excessive lipogenesis in hepatocytes.^10^^,^^11^ In line with the selective activation of LXR in macrophages,^14^ SH42 did not change the overall hepatic expression of lipogenic genes in E3L.CETP mice. These data also support our previous observations that SH42 does not induce lipogenesis in E3L.CETP mice fed a high-fat high-cholesterol diet to induce metabolic dysfunction-associated steatohepatitis, and in fact markedly reduces both liver steatosis and inflammation.^17^ Interestingly, the expression of hepatic *Cpt1* which is involved in fatty acid oxidation was decreased by SH42 treatment. The effect of SH42/desmosterol on fatty acid oxidation has not been investigated in detail. Given that the interaction between fatty acid and cholesterol metabolism has been widely reported^29^^,^^30^ and increasing hepatic cholesterol by dietary cholesterol supplementation has been shown to reduce *Cpt1* expression, we speculate that the upregulated *Cpt1* may be connected to the decreased hepatic cholesterol levels following SH42 treatment in this study. While desmosterol accumulation in macrophages upon SH42 treatment has previously been described to upregulate the expression of the LXR target genes *Abcg1* and *Abca1* that encode cholesterol transporters,^14^^,^^31^ we did not observe altered *Abcg1* and *Abca1* gene expression in the liver here. This may be explained by the modest contribution of monocytes and macrophages to the total cell count in the liver, with hepatocytes predominating.

It is well-established that intracellular cholesterol levels can determine hepatic LDLR expression through a negative feedback mechanism.^32^^,^^33^^,^^34^ Surprisingly, a decrease in hepatic cholesterol was observed in E3L.CETP mice coincided with a tendency toward decreased hepatic LDL receptor protein abundance with SH42 treatment. Some studies have demonstrated that desmosterol may partly replace cholesterol in certain physiological processes.^35^^,^^36^ A study found that desmosterol has a regulatory feedback role and can reduce LDL receptor protein levels, in a manner similar to that of supplementary cholesterol.^37^ Thus, accumulated desmosterol by SH42 treatment may decrease the abundance of LDL receptors. This also suggests that SH42 may reduce the LDL receptor-mediated hepatic uptake of ApoE-containing TG-rich lipoprotein remnants. However, SH42 did not decrease the hepatic uptake of radioactively labeled TG-rich lipoprotein-like particles in this study. Of note, the kinetic study with radiolabeled particles was performed 4 h after the last dosing of SH42, possibly missing a significant effect on hepatic lipoprotein remnant uptake. Nonetheless, the fact that SH42 increases desmosterol levels by blocking its conversion into cholesterol seems to be the most likely reason for the decrease in hepatic cholesterol content, while this effect is not strong enough to influence circulating cholesterol levels, especially in the context of a cholesterol-rich diet. Taken together, DHCR24 inhibition by SH42 does not induce hepatic lipogenesis and in fact lowers hepatic cholesterol, presumably as a consequence of macrophage-specific LXR activation and generally lowered cholesterol synthesis.

While we previously showed that SH42 was very effective in attenuating experimental peritonitis^16^ and metabolic dysfunction-associated steatohepatitis,^17^ we did not observe an effect of SH42 treatment on atherosclerotic lesion area, severity or markers of plaque stability either in E3L.CETP mice, a model for lipid-driven atherosclerosis, or in LDLr-KO mice, a model of inflammation-driven atherosclerosis. This was quite surprising, as desmosterol depletion induced by overexpression of DHCR24 in myeloid cells in male LDLr-KO mice fed a diet containing 21% fat and 1.25% cholesterol was demonstrated to aggravate atherosclerosis by increasing macrophage-driven inflammation.^31^ Circulating monocytes and monocyte subsets usually strongly correlate with atherosclerotic progression in mice and humans.^38^^,^^39^ Although the exact roles of monocyte subtypes in atherosclerosis development are still under debate,^40^^,^^41^ NCMs and IMs have been described as more inflammatory than CMs due to their higher pro-inflammatory cytokine production.^42^^,^^43^ In this study, while CM levels were unaffected, SH42 reduced the proportion of circulating NCMs in LDLr-KO mice where IMs tended to be increased. However, the area of ICAM-1 which is responsible for the recruitment of circulating monocytes in atherosclerotic lesions was not affected. Interestingly, SH42 treatment largely increased the lesion content of smooth muscle cells that contribute to stabilizing the plaque by generating the extracellular matrix and supporting the development of fibrous caps.^44^ Nonetheless, the lesion stability index was overall not altered due to unchanged macrophage and collagen content of the lesions. Here, it may be possible that we did not obtain a sufficiently high level of desmosterol specifically in macrophages within atherosclerotic plaques to exert a measurable effect on atherosclerosis development. Also, it has been described that in the transition from macrophage to foam cell, DHCR24 is strongly suppressed.^13^ This could explain why no added benefits of DHCR24 inhibition were observed in the current study.

As mentioned in the results section, the proximity of *DHCR24* and *PCSK9* at the same locus does not rule out a genetic association between *DHCR24* variants locus and CAD in humans. Future studies should thus be performed in humans to investigate the association between *DHCR24* variation and desmosterol levels, and between desmosterol levels and CAD.

In summary, we demonstrated that the DHCR24 inhibitor SH42 induces a profound increase in desmosterol levels in both E3L.CETP mice and LDLr-KO mice without stimulation of lipogenesis or aggravating hyperlipidemia, and even reduces hepatic lipids as shown in E3L.CETP mice. Desmosterol accumulation through SH42 treatment has stronger effects on blood monocyte populations and atherosclerotic lesion composition in LDLr-KO mice than in E3L.CETP mice but in neither mouse model ameliorates atherosclerotic progression.

### Limitations of the study

Firstly, while SH42 has been reported to enhance LXR activation in macrophages, we were unable to evaluate the effect of SH42 on monocytes/macrophages that reside within the lesions in our study due to technical limitations associated with the flow cytometry used to characterize the macrophage subpopulations. Secondly, we observed that SH42 decreases hepatic lipid content, the underlying mechanism of which was not fully elucidated. It is worth exploring the potential therapeutic effects of SH42 on lipid-driven liver diseases such as metabolic dysfunction-associated steatohepatitis further.

## STAR★Methods

### Key resources table

**Table:** 

REAGENT or RESOURCE	SOURCE	IDENTIFIER
**Antibodies**		

α-actin	Dako	Cat# M0851
Anti-goat secondary antibody	ProteinSimple	Cat# DM-002
Anti-rabbit secondary antibody	ProteinSimple	Cat# DM-001
CD11b	Biolegend	Cat# 101251; RRID: AB_11203704
CD11b	eBioscience	Cat# 25-0112-82
CD19	eBioscience	Cat# 11-0191-85
CD19	eBioscience	Cat# 12-0193-83
CD3	Biolegend	Cat# 100237; RRID: AB_2562039
CD45	Biolegend	Cat# 103149; RRID: AB_2564590
Fixable Viability Dye	eBioscience	Cat# 15383562
GAPDH	Cell signaling	Cat# 2118; RRID: AB_561053
HRP-labeled secondary antibody	Vector Laboratories	Cat# MP-7444; RRID: AB_2336530
ICAM-1	Sino Biological	Cat# 50440-R020; RRID: AB_2860508
LDLR	R&D system	Cat# AF2255; RRID: AB_355203
Ly6C	Biolegend	Cat# 128033; RRID: AB_2562351
Ly6C	eBioscience	Cat# 17-59432-82
Ly6G	Biolegend	Cat# 108106; RRID: AB_313342
Ly6G	Biolegend	Cat# 127664; RRID: AB_2860671
Mac-3	BD Pharmingen	Cat# 550292; RRID: AB_393587
NK1.1	Biolegend	Cat# 108727; RRID: AB_2132705
NK1.1	BD Horizon	Cat# 564143; RRID: AB_2738617
Siglec-F	BD Biosciences	Cat# 746668; RRID: AB_2743940
Thy1.2	Biolegend	Cat# 140324; RRID: AB_2566739

**Chemicals, peptides, and recombinant proteins**		

Brilliant Stain Buffer Plus	BD Biosciences	566385
Chlorotrimethylsilane	ThermoFisher	110120010
[^14^C]cholesteryl oleate	American Radiolabeled Chemicals	0689
Desmosterol-d6	Avanti Polar Lipids	700040
EnVision System-HRP Labeled Polymer	Dako	K4001
Erythrocyte lysis buffer	BD Biosciences	555899
Liquid Dab + Substrate Chromogen System	Dako	K3468
Methyl *tert*-butyl ether	Honeywell Riedel-de Haën	650560
*N*-methyl-*N*-trimethylsilyl-trifluoroacetamide	Macherey-Nagel	701270.201
*N*-trimethylsilyl-imidazole	Sigma Aldrich	153583
Protease and phosphatase inhibitor	ThermoFisher	A32959
Sirius Red	Sigma Aldrich	365548-5G
Solvable	PerkinElmer	6NE9100
True-Stain Monocyte Blocker	Biolegend	426102
Tri[^3^H]oleate	American Radiolabeled Chemicals	0199
TriPure RNA Isolation Reagent	Sigma Aldrich	11667157001
Ultima Gold liquid scintillation cocktail	PerkinElmer	6013321

**Critical commercial assays**		

Triglyceride assay kit	Roche Diagnostics	10166588130
BCA Protein assay kit	ThermoFisher	A55860
Cholesterol assay kit	Roche Diagnostics	11489232216
Phospholipids assay kit	Instruchemie	3009

**Deposited data**		

Genome-wide association study (GWAS) of the combined cross-ancestry meta-analysis on CAD	Aragam KG, Nat Genet, 2022	Aragam et al.^26^

**Experimental models: Organisms/strains**		

Mouse: APOE∗3-Leiden.CETP	Leiden University Medical Center	N/A
Mouse: LDLr-KO (B6.129S7-Ldlrtm1Her/J)	Jackson Laboratory	002207

**Oligonucleotides**		

Primer sequences	Biolegio, see Table S1	N/A

**Software and algorithms**		

GraphPad Prism v9.3	GraphPad Software Inc.	https://www.graphpad.com/updates
Compass for Simple Western v4.0.0	Bio-Techne	https://www.bio-techne.com/resources/instrument-software-download-center/compass-software-simple-western
FlowJoTM v10.8	BD Biosciences	https://www.flowjo.com/solutions/flowjo/downloads/previous-versions
ImageJ software v1.52a		https://github.com/imagej/ImageJ
RandoMice v1.1.6	van Eenige R, PLoS One, 2020	van Eenige et al.^45^
SpectroFlo v3.0	Cytek Biosciences	https://cytekbio.com/pages/spectro-flo

### Resource availability

#### Lead contact

Further information and requests for resources should be directed to and will be fulfilled by the lead contact, Milena Schönke (m.schoenke@lumc.nl).

#### Materials availability

This study did not generate unique reagents.

#### Data and code availability

• All data reported in this paper will be shared by the lead contact upon request.
• No new code was generated in this study.
• Any additional information required to reanalyze the data reported in this paper is available from the lead contact upon request.

### Experimental model and study participant details

#### Animals and treatments

In experiments 1 and 2, female E3L.CETP mice (8–12 weeks of age) were used. These mice were generated by crossbreeding hemizygous APOE∗3-Leiden (E3L) mice with mice expressing human cholesteryl ester transfer protein (CETP), both on a C57BL/6J background, as described previously.^46^ The mice were fed a Western-type diet (Ssniff Spezialdiäten GmbH, Germany) containing 16% fat and 0.15% cholesterol. Following a 3-week dietary run-in period to induce hyperlipidemia, mice were block randomized using RandoMice v1.1.6^45^ into two groups balanced for plasma lipid levels and body composition and treated either with the DHCR24 inhibitor SH42 (0.5 mg dissolved in 7.5 μL ethanol and 7.5 μL Cremophor EL in 135 μL saline) or vehicle 3 times per week for 6 weeks in experiment 1 (n=11-12 per group) and for 15 weeks in experiment 2 (n=16 per group) *via* intraperitoneal injection.

In experiment 3, male LDLr-KO mice (8–12 weeks of age) on a C57BL/6J background were used (B6.129S7-Ldlrtm1Her/J; Jackson Laboratory, USA). After block-randomization into two groups (n=13-15 per group) as described above, the mice were fed a Western-type diet (Ssniff Spezialdiäten GmbH, Germany) containing 16% fat and 0.25% cholesterol, and treated with either 0.5 mg DHCR24 inhibitor SH42 or vehicle and 3 times per week for 13 weeks *via* intraperitoneal injection. All mice were group housed under a 12-hour light-dark cycle and had free access to water and food unless indicated otherwise. All mouse experiments were reviewed by the Animal Welfare Body Leiden and executed under a license granted by the Central Authority for Scientific Procedures on Animals (CCD) under the license number AVD11600202010187 in accordance with the Dutch Act on Animal Experimentation and EU Directive 2010/63/EU. The experiments were executed at Leiden University Medical Center.

### Method details

#### Body weight, body composition and food intake

Mice were weighed on a weighing scale, body composition was determined by an EchoMRI-100 analyzer (EchoMRI, USA), and food intake was determined by monitoring the food consumption (per cage) biweekly in experiment 1 and every 4 (or 6) weeks in experiment 2 and 3.

#### Plasma lipid levels

Blood was collected biweekly in experiment 1, and every 4 (or 6) weeks in experiment 2 and 3 *via* a tail vein cut after 4 hours of fasting. Plasma was obtained through centrifugation and TG and TC levels were quantified using enzymatic kits (Roche Diagnostics, Germany).

#### *In vivo* plasma decay and organ uptake of triglyceride-rich lipoprotein-like particles

In experiment 1, after 6 weeks of treatment, TG-rich lipoprotein-like particles double-labeled with glycerol tri[^3^H]oleate (American Radiolabeled Chemicals, USA) and [^14^C]cholesteryl oleate (American Radiolabeled Chemicals, USA) were prepared as previously described^47^ and injected into the tail vein of the mice (1.0 mg TG in 200 μL saline per mouse). Blood samples were drawn from the tail vein at 2, 5, 10, and 15 min after the particle injection. Subsequently, mice were killed by CO_2_ inhalation and perfused with ice-cold PBS before organs were collected. Plasma samples collected following the particle injection and tissue samples that had been dissolved overnight at 55°C in Solvable (PerkinElmer, The Netherlands) were mixed with Ultima Gold liquid scintillation cocktail (PerkinElmer, The Netherlands). ^3^H and ^14^C activity in the samples (disintegrations per minute; dpm) were quantified using a Tri-Carb 2910TR low-activity liquid scintillation analyzer (PerkinElmer, The Netherlands). Decay of ^3^H and ^14^C radioactivity in plasma was expressed as the percentage of injected radioactive dose. Uptake of ^3^H and ^14^C radioactivity by the organs was expressed as the percentage of injected radioactive dose per gram tissue.

#### Desmosterol quantification

In experiment 1 and 3, after respectively 6 and 13 weeks of treatment, total desmosterol levels in the liver and plasma were quantified *via* gas chromatography-mass spectrometry (GC-MS) analysis as previously reported^48^^,^^49^ with minor modifications. Briefly, 12.5 μL liver homogenates (∼2.5 mg tissue) or 5-10 μL plasma samples were mixed with 70 μL ethanol and 10 μL internal standard mixture containing 20 μg/mL desmosterol-d6 (Avanti Polar Lipids, USA) in ethanol. After adding 10 μL aqueous NaOH solution (10 M), the mixtures were flushed with nitrogen gas and then saponified for 1 hour at 70°C. Subsequently, sterols were extracted with methyl *tert*-butyl ether (Honeywell Riedel-de Haën, Germany) and dried with sodium sulfate. Next, the extracts were centrifuged for 5 min at 10,000 rcf and the supernatants were dried with nitrogen gas. Finally, the samples were reconstituted in a mixture of *N*-methyl-*N*-trimethylsilyl-trifluoroacetamide (Macherey-Nagel, Germany) containing 1% Chlorotrimethylsilane (ThermoFisher, USA) and *N*-trimethylsilyl-imidazole (Sigma Aldrich, USA). An Agilent 8890 GC system coupled with an Agilent 5977B MS was used for desmosterol quantification. The injector was held at 300°C and 1 μL sample was injected splitless. Sterols were separated on a VF-5ms column (30 m × 0.25 mm × 0.25 μm) using the following temperature gradient: 1 min at 50°C, linear increase at 50°C/min to 260°C, linear increase at 4 °C/min to 310°C, held for 2.3 min at 310°C. Helium was used as carrier gas at a constant flow rate of 1.40 mL/min. The transfer line was set at 280°C, the source at 230°C and the quadrupole at 150°C. The MS was operated in single ion monitoring mode (333.3 and 327.2 were used as quantifiers for desmosterol-d6 and desmosterol, respectively). Desmosterol was quantified using external calibration.

#### Hepatic lipid content and histology

In experiment 1, after 6 weeks of treatment, liver samples were collected and hepatic lipids were extracted from snap-frozen liver samples according to a modified protocol from Bligh and Dyer.^50^ Liver TG and TC (both Roche Diagnostics, Germany) and PL (Instruchemie, The Netherlands) were measured *via* enzymatic kits. Protein concentrations were measured using a BCA Protein assay kit (ThermoFisher, USA). Hepatic lipid content was expressed as nmol per mg protein. Liver samples were fixated in phosphate-buffered formaldehyde, embedded in paraffin and sectioned at 5 μm thickness. The tissue sections were stained with hematoxylin-eosin (HE) and areas of lipid accumulation (i.e., unstained areas) were quantified using ImageJ software v1.52a.

#### Gene expression analysis

In experiment 1, after 6 weeks of treatment, total RNA was isolated from snap-frozen liver samples with TriPure RNA Isolation Reagent (Sigma Aldrich, USA). Isolated RNA was reverse-transcribed into cDNA using Moloney murine leukemia virus reverse transcriptase (Promega, USA). Quantitative real-time PCR was performed using SYBR green (Promega, USA) on a CFX96 machine (Bio-Rad, USA). mRNA expression levels were normalized to Beta-actin (*Actb*) and glyceraldehyde-3-phosphate dehydrogenase (*Gapdh)* mRNA expression and expressed as fold change compared with the vehicle group using the ΔΔCt method. The primer sequences are listed in Table S1.

#### Hepatic LDL receptor protein level

In experiment 1, after 6 weeks of treatment, liver samples were collected and lysed in Radioimmunoprecipitation assay buffer (ThermoFisher, USA) containing protease and phosphatase inhibitor (ThermoFisher, USA), and protein was extracted as described previously.^51^ Protein concentration in the lysate was determined using a BCA Protein assay kit (ThermoFisher, USA). Subsequently, Western blots for LDLR and GAPDH were performed separately in 25 capillary cartridges (ProteinSimple, USA) according to the 12–230 kDa Jess and Wes Separation Module protocol. In short, the protein samples mixed with a fluorescent master mix were heated at 95°C for 5 min. Protein samples, goat IgG anti-mouse LDLR (R&D System, USA) or rabbit IgG anti-mouse GAPDH (Cell Signaling, USA), and anti-goat or anti-rabbit secondary antibodies (ProteinSimple, USA) were then loaded in Wes assay plates according to the kit manual instructions. The separation and immunodetection of LDLR and GAPDH proteins were performed by the Wes Western blotting system and the results were analyzed by Compass for Simple Western v4.0.0 (ProteinSimple, USA). Data are shown as fold change from control.

#### Blood leukocyte isolation and flow cytometry analysis

In experiment 2, after 15 weeks of treatment, 8 mice per group were selected, based on having body weight, lean mass and fat mass close to the group mean, and 12-hour-fasted blood was collected from the retro-orbital plexus just after the mice were killed by CO_2_ inhalation. In experiment 3, after 12 weeks of treatment, 4-hour-fasted blood was collected *via* a tail vein cut. Obtained blood samples were lysed for 20 min at room temperature using an erythrocyte lysis/fixation solution (BD Biosciences, USA). Leukocytes were then centrifuged at 596 rcf for 5 minutes at 4°C, reconstituted in PBS, and washed three times in PBS. Isolated leukocytes were treated with a cocktail of antibodies (details are provided in Tables S2 and S3) in PBS containing 2 mM EDTA, 0.5% BSA, True-Stain monocyte blocker (Biolegend, USA) and Brilliant Stain Buffer Plus (BD Biosciences, USA) for 30 minutes at 4°C, as previously reported.^52^ After staining, samples were analyzed by spectral flow cytometry using a Cytek Aurora Spectral flow cytometer (Cytek Biosciences, The Netherlands) or a Cytoflex S (Beckman Coulter, Woerden, the Netherlands). SpectroFlo v3.0 (Cytek Biosciences, The Netherlands) and FlowJoTM v10.8 software (BD Biosciences, USA) were used for spectral unmixing of the flow cytometry data and gating of the data, respectively. A representative gating strategy is shown in Figures S3 and S4.

#### Atherosclerosis quantification

In experiment 2 and 3, after respectively 15 and 13 weeks of treatment, hearts were collected, fixed in phosphate-buffered formaldehyde, and embedded in paraffin. After cross-sectioning (5 μm) throughout the aortic root area, 4 consecutive sections per heart in 50 μm intervals starting at the opening of the aortic valves were used for the quantification of atherosclerotic lesions. Cross-sections were stained with HPS to determine lesion areas. According to the guidelines of the American Heart Association adapted for mice,^53^ lesions were categorized into mild lesions (type I-III, with foam cells in the intima/media or/and the presence of a fibrotic cap) or severe lesions (type IV-V, with fibrosis and progressive lesion infiltrating into the media or/and the presence of cholesterol clefs/mineralization/necrosis). Smooth muscle cells were stained using an anti-α-actin antibody (Dako, Denmark) and a secondary antibody EnVision System-HRP Labeled Polymer (Dako, Denmark) that was visualized by Liquid DAB + Substrate Chromogen System (Dako, Denmark). Collagen was stained with Sirius Red (Sigma Aldrich, USA). Macrophages were stained with an anti-Mac-3 antibody (BD Pharmingen, USA), an HRP-labeled secondary antibody and a peroxide substrate (Vector Laboratories, USA). In experiment 3, Intercellular adhesion molecule 1 (ICAM-1) area was stained using an anti-ICAM-1 antibody (Sino Biological, USA) and a secondary antibody EnVision System-HRP Labeled Polymer (Dako, Denmark) that was visualized by Liquid DAB + Substrate Chromogen System (Dako, Denmark). Lesion areas and the areas of smooth muscle cells, collagen, macrophages and ICAM-1 were quantified using ImageJ software v1.52a.

#### Genetic association with coronary artery disease in humans

We used summary statistics from the genome-wide association study (GWAS) of the combined cross-ancestry meta-analysis on CAD published by Aragam et al.^26^ which compiles data from 181,522 CAD patients among 1,165,690 participants. The data were downloaded from the GWAS catalog: https://www.ebi.ac.uk/gwas/publications/36474045. Genetic associations within the *DHCR24* locus were plotted with LocusZoom.^54^

### Quantification and statistical analysis

Data were analyzed by GraphPad Prism Software, v9.3. Differences between the two groups were compared using unpaired two-tailed Student’s t-tests for parametric data or Mann Whitney U test for nonparametric data. For experiments involving repeated measurements in the same animal, two-way repeated-measures ANOVA and Bonferroni post hoc analyses were performed. Data are shown as mean ± SEM. *P* < 0.05 was considered significant.
