# Supplementary material for: DHCR24 inhibitor SH42 increases desmosterol without preventing atherosclerosis development in mice
Source: iScience. 2024 Apr 26;27(6):109830. doi: 10.1016/j.isci.2024.109830 (PMC11103367; doi:10.1016/j.isci.2024.109830)
Supplement: Document S1. Figures S1–S4 and Tables S1–S3 [file mmc1.pdf]

iScience, Volume *27*

Supplemental information

DHCR24 inhibitor SH42 increases desmosterol without preventing atherosclerosis development in mice

Xiaoke Ge, Bram Slütter, Joost M. Lambooij, Enchen Zhou, Zhixiong Ying, Ceren Agirman, Marieke Heijink, Antoine Rimbert, Bruno Guigas, Johan Kuiper, Christoph Müller, Franz Bracher, Martin Giera, Sander Kooijman, Patrick C.N. Rensen, Yanan Wang, and Milena Schönke

Table S1. Primer sequences for quantitative real-time PCR, related to Figure 2C and STAR Methods

	Primers	
Gene	Forward (5'- 3')	Reverse (5'- 3')
Abca1	CCCAGAGCAAAAAGCGACTC	GGTCATCATCACTTTGGTCCTTG
Abcg1	AGGTCTCAGCCTTCTAAAGTTCCTC	TCTCTCGAAGTGAATGAAATTTATCG
Acc1	AACGTGCAATCCGATTTGTT	GAGCAGTTCTGGGAGTTTCG
Actb	AACCGTGAAAAGATGACCCAGAT	CACAGCCTGGATGGCTACGTA
Apob	GCCCATTGTGGACAAGTTGATC	CCAGGACTTGGAGGTCTTGGA
Cpt1	GAGACTTCCAACGCATGACA	ATGGGTTGGGGTGATGTAGA
Fasn	GCGCTCCTCGCTTGTCGTCT	TAGAGCCCAGCCTTCCATCTCCTG
Fdft1	TGCCTCAGAGTTTGAAGACCCCAT	TCCTGAGGCCAAAACTTCCTTCCT
Fdps	ATGGAGATGGGCGAGTTCTTC	CCGACCTTTCCCGTCACA
Gapdh	GGGGCTGGCATTGCTCTCAA	TTGCTCAGTGTCCTTGCTGGGG
Hmgcr	CCGGCAACAACAAGATCTGTG	ATGTACAGGATGGCGATGCA
Lss	AGGAGCACGTTTCTCGGATCAA	AGGGCAGAAACTCAGGTCTGTG
Mttp	CTCTTGGCAGTGCTTTTTCTCT	GAGCTTGTATAGCCGCTCATT
Ppara	ATGCCAGTACTGCCGTTTTC	GGCCTTGACCTTGTTCATGT
Sqle	CCAACTCAATGGGTCTGTTCCT	TGGCTTAGCAAAGTCTTCCAACT
Srebf1c	AGCCGTGGTGAGAAGCGCAC	ACACCAGGTCCTTCAGTGATTTGCT
Srebf2	TGAAGCTGGCCAATCAGAAAA	ACATCACTGTCCACCAGACTGC

Abca1, ATP binding cassette subfamily A member 1; *Abcg1*, ATP binding cassette subfamily G member 1; *Acc1*, acetyl coenzyme A carboxylase 1; *Actb*, Beta-actin; *Apob,* apolipoprotein B; *Cpt1*, carnitine palmitoyl transferase 1; *Fasn*, fatty acid synthase; *Fdft1*, farnesyl-diphosphate farnesyltransferase 1; *Fdps*, farnesyl diphosphate synthetase; *Gapdh*, glyceraldehyde-3-phosphate dehydrogenase; *Hmgcr*, 3-hydroxy-3-methylglutaryl coenzyme A; *Lss*, lanosterol synthase; *Mttp*, microsomal triglyceride transfer protein; *Ppara*, peroxisome proliferator-activated receptor alpha; *Sqle*, squalene epoxidase; *Srebf1c*, sterol regulatory element-binding factor 1c; *Srebf2*, sterol regulatory element-binding factor 2.

Table S2. Antibodies used for flow cytometry in blood leukocytes of APOE*3-Leiden.CETP mice, related to Figure 3C

Target	Clone	Conjugate	Source	Catalog number
CD3	17A2	BV605	Biolegend	100237
CD11b	M1/70	PE-Cy7	eBioscience	25-0112-82
CD19	MB19-1	FITC	eBioscience	11-0191-85
CD45	30-F11	BV785	Biolegend	103149
Ly6C	HK1.4	BV510	Biolegend	128033
Ly6G	1A8	Spark Blue 550	Biolegend	127664
NK1.1	PK136	PerCP-Cy5.5	Biolegend	108727
Siglec-F	E50-2440	BV480	BD Biosciences	746668

Table S3. Antibodies used for flow cytometry in blood leukocytes of LDL receptor knockout mice, related to Figure 5B

Target	Clone	Conjugate	Source	Catalog number
CD11b	M1/70	BV420	Biolegend	101251
CD19	eBio1D3	Pe	eBioscience	12-0193-83
Fixable Viability Dye	NA	eFluor780	eBioscience	15383562
Ly6C	HK1.4	APC	eBioscience	17-59432-82
Ly6G	D7	FITC	Biolegend	108106
NK1.1	PK136	BV650	BD Horizon	564143
Thy1.2	53-2.1	Pe-Cy	Biolegend	140324

Fig. S1. DHCR24 inhibitor SH42 does not affect the body weight, body composition or plasma lipid levels in APOE*3-Leiden.CETP mice with 15 weeks of treatment, related to Figure 3

Throughout 15 weeks of treatment, cumulative food intake (**D**), body weight (**E**), fat mass (**F**) and lean mass (**G**) were determined at week 4, 8 and 14. After 15 weeks of treatment, the weight of various organs (**H**) was determined. Fasting plasma triglycerides (TG) (**I**) and total cholesterol (TC) (**J**) were measured at week 4, 8 and 14. gWAT, gonadal white adipose tissue; iBAT, interscapular brown adipose tissue; sBAT, subscapular brown adipose tissue; sWAT, subcutaneous white adipose tissue. Data are shown as mean ± SEM. A: n=5 cages per group. B-G: n=15-16 mice per group. A-C, F and G: data were analyzed by two-way repeated-measures ANOVA and Bonferroni post hoc analysis. E: data were analyzed by unpaired two-tailed Student's t-test. *P<0.05.

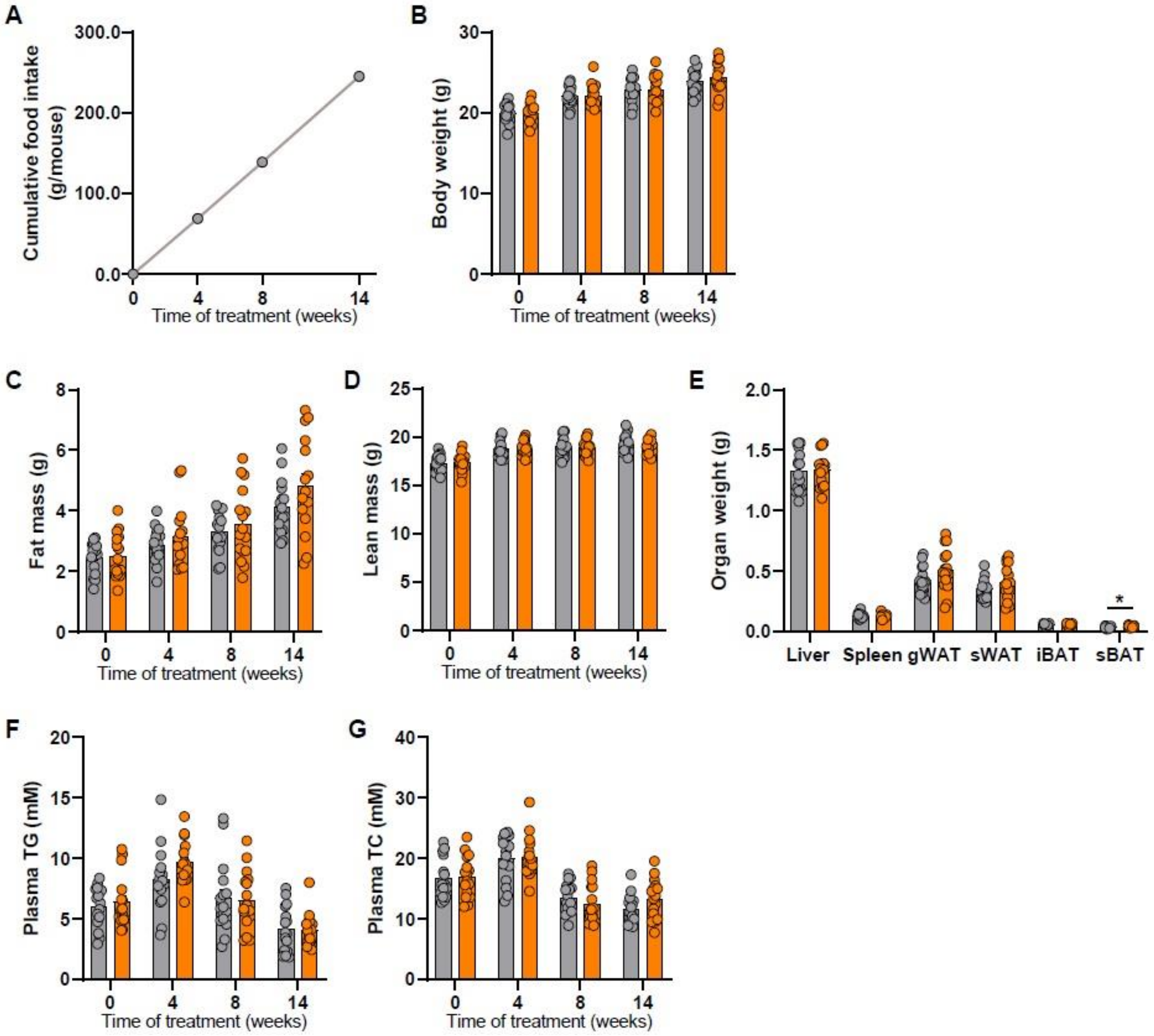

Fig. S2. Genetic association of *DHCR24* variants with coronary artery disease in humans

Locus zoom showing genetic associations of variants in the *DHCR24* locus (1p32.3, GRCh37); chr1:54,815,386-55,852,855 (Upper panel) and chr1:55,265,300-55,402,921 (lower panel) with coronary artery disease. The data comes from the genome-wide association study for coronary artery disease published by Aragam *et al*.[1] comprising 181,522 cases among 1,165,690 participants of predominantly European ancestry. Each symbol corresponds to individual genetic variants in the locus. The purple diamond corresponds to the variant with the lowest *P* value in the window. The color code represents the linkage disequilibrium (LD) in r^2, with the top associated variant (color scale in the top left-hand corner). The grey dotted line depicts the genome-wide significance *P* value threshold of 5×10^{-8}.

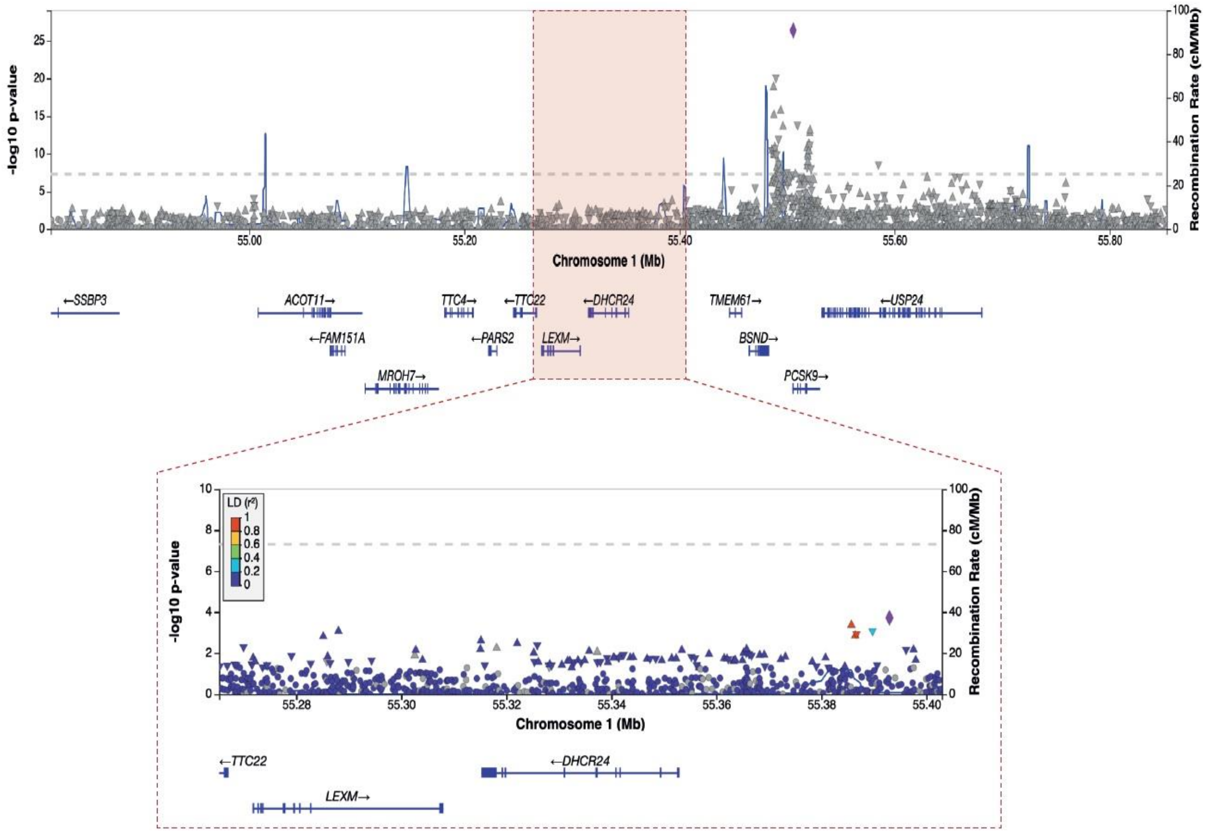

Fig. S3. Gating strategy for flow cytometry in blood leukocytes of APOE*3-Leiden.CETP mice, related to Figure 3C

APOE*3-Leiden.CETP (E3L.CETP) mice were fed a Western-type diet containing 16% fat and 0.15% cholesterol and received intraperitoneal injections with either SH42 (0.5 mg/mouse) or vehicle 3 times per week. After 15 weeks of treatment, blood samples were collected for flow cytometry. A representative gating strategy is presented.

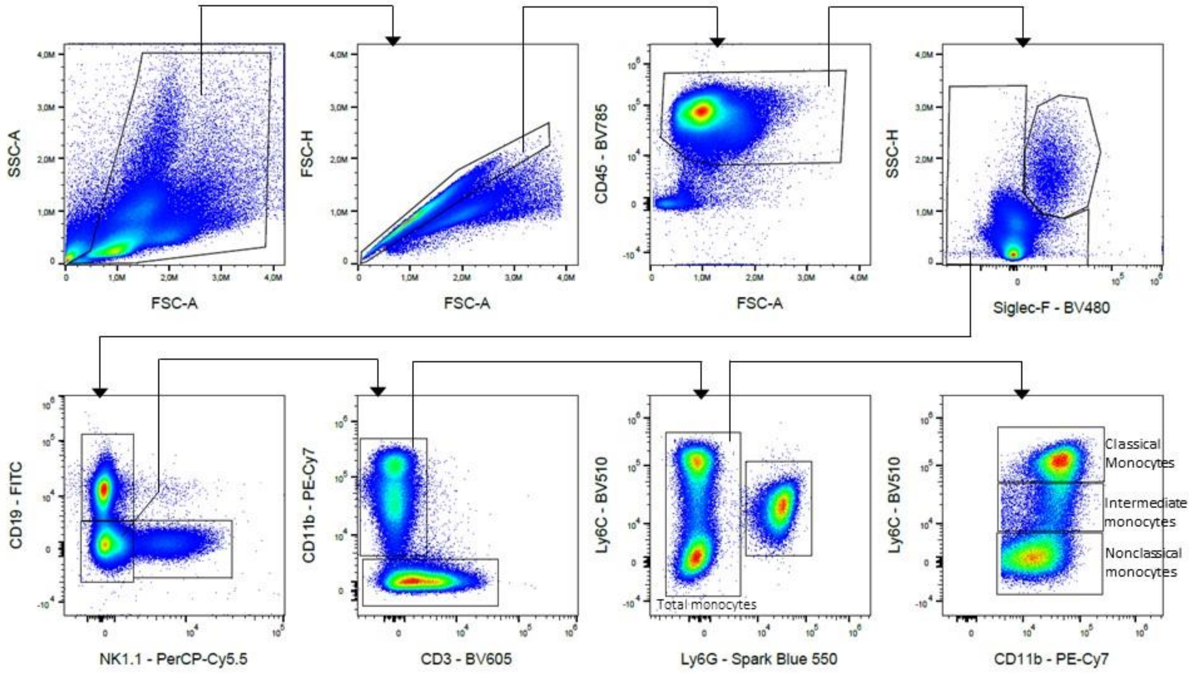

Fig. S4. Gating strategy for flow cytometry in blood leukocytes of LDL receptor knockout mice, related to Figure 5B

LDL receptor knockout (LDLr-KO) mice were fed a Western-type diet containing 16% fat and 0.25% cholesterol and received intraperitoneal injections with either SH42 (0.5 mg/mouse) or vehicle 3 times per week. After 12 weeks of treatment, blood samples were collected for flow cytometry. A representative gating strategy is presented.

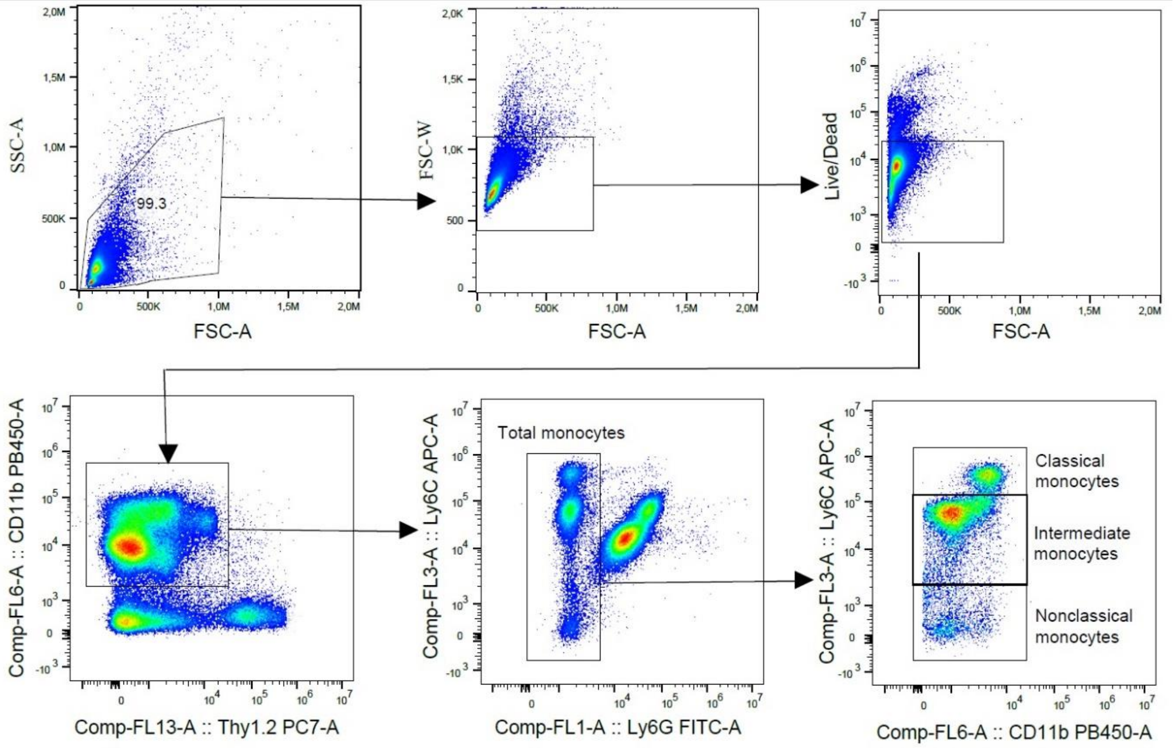

Supplemental reference

1. Aragam, K.G., Jiang, T., Goel, A., Kanoni, S., Wolford, B.N., Atri, D.S., Weeks, E.M., Wang, M., Hindy, G., Zhou, W., et al. (2022). Discovery and systematic characterization of risk variants and genes for coronary artery disease in over a million participants. Nat Genet *54*, 1803-1815. 10.1038/s41588-022-01233-6.
